# Label-Free Time-Gated Luminescent Detection Method for the Nucleotides with Varying Phosphate Content

**DOI:** 10.3390/s18113989

**Published:** 2018-11-16

**Authors:** Kari Kopra, Tanja Seppälä, Dana Rabara, Maria Abreu-Blanco, Sakari Kulmala, Matthew Holderfield, Harri Härmä

**Affiliations:** 1 Materials Chemistry and Chemical Analysis, University of Turku, Vatselankatu 2, 20500 Turku, Finland; tatese@utu.fi (T.S.); harri.harma@utu.fi (H.H.); 2 NCI-RAS Initiative, Cancer Research Technology Program, Frederick National Laboratory for Cancer Research, Leidos Biomedical Research, Frederick, MD 21702, USA; dana.rabara@nih.gov (D.R.); maria.abreublanco@nih.gov (M.A.-B.); matthew.holderfield@nih.gov (M.H.); 3 Laboratory of Analytical Chemistry, Department of Chemistry, Aalto University, P.O. Box 16100, 00076 Aalto, Finland; sakari.kulmala@aalto.fi

**Keywords:** nucleotide triphosphate, label-free, K-Ras, apyrase, terbium

## Abstract

A new label-free molecular probe for luminescent nucleotide detection in neutral aqueous solution is presented. Phosphate-containing molecules, such as nucleotides possess vital role in cell metabolism, energy economy, and various signaling processes. Thus, the monitoring of nucleotide concentration and nucleotide related enzymatic reactions is of high importance. Two component lanthanide complex formed from Tb(III) ion carrier and light harvesting antenna, readily distinguishes nucleotides containing different number of phosphates and enable direct detection of enzymatic reactions converting nucleotide triphosphate (NTP) to nucleotide di/monophosphate or the opposite. Developed sensor enables the detection of enzymatic activity with a low nanomolar sensitivity, as highlighted with K-Ras and apyrase enzymes in their hydrolysis assays performed in a high throughput screening compatible 384-well plate format.

## 1. Introduction

Phosphate-containing molecules such as nucleotides have vital roles in living cells. ATP is the universal energy molecule and signal mediator, while the main function of GTP is in signaling and it has only a secondary role in energy transduction [1,2,3]. Other nucleotide triphosphates (NTPs) have more specified roles in various enzymatic reaction, DNA synthesis, replication, and cell division [4,5]. For these reasons, there has been continuous interest in nucleotide sensors, especially for ATP sensing [6,7,8,9]. During recent years, nucleotide sensors based on chromatographic methods, bioluminescence, and luminescence have been developed [6,7,8,9,10]. These methods have a large variation in sensitivity, simplicity, and robustness. For NTP sensing, bioluminescence methods based on luciferin-luciferase reaction are currently the method of choice mostly due to the high sensitivity [9].

Luminescent molecular probes are widely used in academia and industry, meeting the needs for affordable, non-radioactive, and simple tools also suitable for high throughput screening (HTS) applications [11]. Lanthanide ions, such as Eu(III) and Tb(III), are poorly luminescent, but in combination with protective chelating structure and light harvesting antenna, these metal complexes have an intense long-lived millisecond scale luminescence [12]. Luminescence of such metal complex probes can be readily modulated through the environment and complex forming agents often leading to luminescence quenching. This property has been reported to enable detection of various targets such as food, steroids, and biologically significant nucleotides [13,14,15,16,17]. Label-free sensors provide the simplest tool for the detection of phosphate-containing molecules e.g., nucleotides. Label-free assays can be performed without specific binding reagent, such as aptamer or antibody, but these methods are often limited in sensitivity and also possess poor functionality in neutral aqueous solutions [18]. Lanthanide-based methods have also been rarely developed and validated to study phosphate-containing molecules in real biochemical environment, reducing the applicability of the methods.

We have previously described antibody-based nucleotide detection system for GTP and cAMP using single-label technique called quenching resonance energy transfer (QRET) [19,20,21,22,23]. Nucleotide detection using antibodies is limited to the affinity of the antibody and detection of multiple different nucleotides requires the usage of different antibodies. Here, we present a label- and antibody-free two-component luminescent terbium(III)-probe for nucleotide triphosphate detection. Sensor forming from two individually non-luminescent parts enables nucleotide triphosphate separation from di- and monophosphates with nanomolar sensitivity in real biochemical context. The functionality of the presented assay was studied in a K-Ras GTP hydrolysis system, performing side-by-side comparison with the specific GTP detection assay utilizing the QRET technique [22,23]. The method was further used in enzymatic apyrase assay, monitoring its hydrolysis activity with ATP and multiple other nucleotides.

## 2. Materials and Methods

### 2.1. Materials

All used phosphate containing molecules (ATP, ADP, AMP-PNP, CTP, cAMP, cGMP, GTP, GDP, GMP, ITP, UTP, UDP, and sodium poly-, tri-, di-, and monophosphates), antenna ligands; antenna 1 (4-hydroxy-6-(trifluoromethoxy)-quinoline-3-carboxylic acid), antenna 2 (4-hydroxy-7-methyl-1,8-naphtyridine-3-carboxylic acid), antenna 3 (1-cyclopropyl-6-fluoro-4-oxo-7-piperazin-1-yl quinoline-3-carboxylic acid), antenna 4 (1-ethyl-1,4-dihydro-7-methyl-4-oxo-1,8-naphthyridine-3-carboxylic acid), antenna 5 (2-methyl-7-oxo-4,7-dihydropyrazolo[1,5-a]pyrimidine-6-carboxylic acid), and antenna 6 (4-oxo-1,4-dihydroquinoline-3-carboxylic acid), TbCl_3_, apyrase (A6410), and DCAI (2-(4,6-dichloro-2-methyl-1h-indol-3-yl)ethanamine) were purchased from Sigma-Aldrich (St. Louis, MO, USA). Diethylenetriamine-N^1^, N^2^, N^3^, N^3^-tetraacetic acid Tb(III)-chelate (Tb(III)-N1-chelate) was obtained from PerkinElmer Life and Analytical Sciences, PerkinElmer Wallac (Turku, Finland). All K-Ras (res. 2-188) proteins (wild-type, G12C, G12D, Q61L, and Q61R), His-SOS^cat^ (res. 564-1049), p120GAP (res. 714-1047), and NF1-333 (res. 1198-1530) were of human origin and produced in *E. coli* [21,22,23]. Eu(III)-GTP was synthetized and conjugated as described elsewhere [22,23,24]. The soluble quencher molecule, MT2, was obtained from QRET Technologies (Turku, Finland), and used according to manufacturer’s instruction. 2A4^GTP^ Fab fragment selection, production, and purification are described elsewhere [23,24]. Black Corning 384 well low volume assay plates (4513) were used in all assays. The small molecule library used was randomly selected part of the DIVERSet™ ChemBridge Diverse Screening Library. Assays were performed in selected assay buffer 1 (20 mM HEPES, pH 7.5, 1 mM MgCl_2_, 0.01% Triton-X 100, 0.005% γ-globulins) if not otherwise mentioned. Apyrase assays were performed in assay buffer 2 (20 mM HEPES, pH 6.5, 1 mM MgCl_2_, 1 mM CaCl_2_, 0.01% Triton-X 100, 0.005% γ-globulins).

### 2.2. Instrumentation

The Eu(III)-GTP purification was carried out using reversed-phase adsorption chromatography, Dionex ultimate 3000 LC system from Thermo Fischer Scientific, Dionex (Sunnyvale, CA, USA), and Ascentis RP-amide C18 column from Sigma-Aldrich, Supelco Analytical. Time-gated luminescence for Eu(III)-chelate was monitored using 615 nm emission and 340 nm excitation wavelengths, and 600 µs delay and 400 µs decay times. Time-gated luminescence for Tb(III)-probes were monitored using 545 nm emission and 330 nm excitation wavelengths, 100 µs delay, and 200 µs decay times. All measurements were performed using a standard microtiter plate reader developed by Labrox Ltd. (Turku, Finland). Pre-plating of the library compounds was performed with an Echo 550 Liquid Handler (Labcyte Inc., San Jose, CA, USA). Emission and excitation spectrums were measured using Varian Cary Eclipse fluorescence spectrophotometer (Agilent Technologies, Mulgrave, Australia). Luminescence excitation spectra’s (220-400 nm) for Tb(III)-N1-chelate and Probe 1 were monitored with 5 nm slit, 545 nm emission with 10 nm slit, 100 µs delay, and 100 µs decay. Luminescence emission spectra (450–700 nm) for Probe 1 was monitored with 5 nm slit, 330 nm excitation with 10 nm slit, 100 µs delay, and 100 µs decay. The emission spectra for Tb(III)-N1-chelate was monitored similarly as for Probe 1, but using 240 nm excitation. Emission lifetime for Probe 1 was monitored using 330 ± 20 nm excitation and 545 ± 20 nm emission wavelengths.

### 2.3. Probe 1 Selection

Preselected antennas (antenna 1 to 6) were first tested with TbCl_3_ and Tb(III)-N1 (2.5–50 nM) in various antenna concentrations (1–100 µM). All assays were performed in 10 µL final volume. Using 10 µM GTP (5 µL) as an analyte, we defined maximal signal-to-background (S/B) and signal stability with antennas 1to 6 (7.5 µM), either with TbCl_3_ (7.5 nM) or Tb(III)-N1 (7.5 nM) added as a preformed complex with different antennas (5 µL). Tb(III)-N1 in complex with all the antennas was further used to perform single concentration (750 nM) nucleotide triphosphate (NTP) assay (GTP, ATP, ITP, UTP, and CTP), and thereafter nucleotide titration (0.5–32,000 nM) using various phosphate containing analytes (ATP, ADP, AMP-PNP, CTP, cAMP, cGMP, GTP, GDP, GMP, ITP, UTP, UDP, and sodium poly-, tri-, di-, and monophosphates). Signal stability was monitored performing multiple measurements between 5 min and 120 min. Based on these experiments, we selected antenna 1 (4-hydroxy-6-(trifluoromethoxy)quinoline-3-carboxylic acid) to form the complex with Tb(III)-N1 (Probe 1).

Tb(III)-N1-chelate alone and Probe 1 were further characterized by measuring luminescence spectra in assay buffer 1 (pH 7.5) in the presence of 0–100 µM GTP and with the parameters listed above. Thereafter Probe 1 was monitored in a modified assay buffer 1 (pH 6–8) and in the presence or absence of 10 µM ATP in 500 µL volume.

Probe 1 tolerance for different small molecular ligands was tested using 320 randomly selected library compounds transferred into the assay plates from 10 mM stock solutions (final concentration 20 µM). Assay plate was complemented with 64 dimethyl sulfoxide (DMSO) controls without compound. Full plate was assayed with GTP (1.5 µM) and without GTP in 10 µL final volume and the Z’-factors were calculated.

### 2.4. Label-Free K-Ras Activity Assays

Probe 1 was used in K-Ras (wild-type and four mutants) GTPase cycling assay to monitor GTP hydrolysis. The GTPase cycling assays, 200 nM K-Ras (wild-type, and G12C, G12D, Q61L, and Q61R mutants), SOS^cat^ (200 nM), p120RasGAP or NF1 (100 nM), GTP (1.5 µM), were performed in a 384-well plate using 5 µL volume (Table 1). After 60 min hydrolysis reaction, the detection was performed using Probe 1 in a 10 µL final volume. Tb(III)-signal was monitored at multiple time points (10–90 min). DCAI titration (1.25–500 µM) was performed using 200 nM K-Ras (wild-type or Q61R mutant), SOS^cat^ (200 nM), p120RasGAP (100 nM), and GTP (1.5 µM). DCAI was added in 1 µL volume (2% DMSO in final volume) and 60 min hydrolysis reaction was performed in 5 µL volume as previously. After incubation, hydrolysis was monitored using Probe 1 in a 10 µL final volume and the time-gated Tb(III)-luminescence signal was monitored as previously. The reproducibility of the GTPase cycling assay was confirmed using K-Ras wild-type and comparing it to K-Ras mutants G12D and Q61R. Assay was performed using 24 replicate reactions supplemented with SOS^cat^ and p120RasGAP. Tb(III)-signals were measured 60 min after Probe 1 addition, reaching the 10 µL final volume. Results were used to calculate Z’-factor.

We subsequently performed GTP association assay using 500 nM K-Ras (wild-type, G12C, and Q61R), SOS^cat^ (250 nM), and GTP (500 nM) (Table 1). Association was performed in 6 µL volume, and after 20 min incubation, the detection was performed using Probe 1 (10 µL final volume). Similarly, DCAI titration (1.25–500 µM) was performed using 500 nM K-Ras (wild-type or Q61R mutant), SOS^cat^ (250 nM), and GTP (500 nM) in 7 µL volume. DCAI was added in 1 µL volume (2% DMSO in final volume). Probe 1 was added in 3 µL to reach the final 10 µL volume. In both assays, time-gated Tb(III)-signals were monitored at multiple time points between 10 min and 90 min.

### 2.5. QRET-Based Control Assay for GTPase Cycling and GTP Association Monitoring

The competitive QRET based GTPase cycling assay was used as a control for the assay performed with Probe 1 [23]. Assays with K-Ras wild-type and mutants (G12C, G12D, Q61L, and Q61R) were performed using same assay conditions listed above and used in case of Probe 1 (Table 1). Enzymatic assays were performed in 5 µL, and time-gated Eu(III)-signals were monitored after QRET detection component (7.5 nM Eu(III)-GTP, 12 nM 2A4^GTP^, and 1.8 µM MT2) addition (10 µL total volume). In DCAI titration (1.25–500 µM) the DMSO concentration was 2% in final 10 µL volume. Time-gated Eu(III)-signal was monitored at multiple time points between 10 min and 90 min.

The Eu(III)-GTP association assay was used as a control assay for label-free GTP association detection using Probe 1 [22]. Eu(III)-GTP association assay was performed in 10 µL volume using 200 nM K-Ras (wild-type, and mutants G12C, G12D, Q61L, and Q61R), SOS^cat^ (200 nM), Eu(III)-GTP (10 nM), and MT2 (1.5 µM) (Table 1). All components were added in 2.5 µL volumes and Eu(III)-signals were monitored between 10 min and 90 min. In DCAI titration (1.25–500 µM) the same protocol was applied and the DMSO concentration was 2% in the final 10 µL volume.

### 2.6. Label-Free Apyrase ATPase Activity Monitoring

Probe 1 was used in ATP hydrolysis monitoring using potato apyrase (ATPase) as a model enzyme. Apyrase assay was performed in assay buffer 2, which was supplemented with 1 mM Ca^2+^ (pH 6.5) (Table 1). First, the apyrase ATPase activity was titrated with 1 or 10 µM ATP or 10 µM AMP-PNP in a 5 µL reaction. After 50 min incubation, the detection was performed using Probe 1 (10 µL final volume). Tb(III)-signals were monitored at multiple time points between 10 min and 60 min. After apyrase titration, the apyrase specificity was tested with the panel of NTPs (ATP, GTP, UTP, CTP, and AMP-PNP) and NDPs (ADP, GDP, and UDP). NTP (10 µM) hydrolysis was performed using 50 µU of apyrase, and with NDPs (50 µM) the used apyrase concentration was 200 µU. After 50 min incubation in 5 µL volume, Probe 1 was added and the time-gated Tb(III)-signal was monitored at multiple time points between 10 min and 60 min.

### 2.7. Data Analysis

In all assays, the signal-to-background ratio (S/B) was calculated as µ_max_/µ_min_, coefficient of variation (CV%) (σ/µ) × 100, and Z’-factor (1 − [(3σ_max_ + 3σ_min_)/(|µ_max_ − µ_min_|)]). In all formulas µ is the mean value, and σ is the standard deviation. All data was analyzed using Origin 8 software (OriginLab, Northampton, MA, USA)*.*

## 3. Results and Discussion

Phosphate-containing molecules, e.g., nucleotides, play important roles in variety of biological processes [1,2,3,4,5]. Nucleotides are involved in energy metabolism and several enzymatic reactions, and the simplest way to efficiently study these reactions is to monitor changes in concentration of nucleotides with varying phosphate content. To address this, we developed label-free sensor for enzymatic nucleotide studies.

### 3.1. Label-Free Terbium(III)-Probe Enables Nanomolar Detection of Phosphate-Containing Molecules

**Figure 1:**
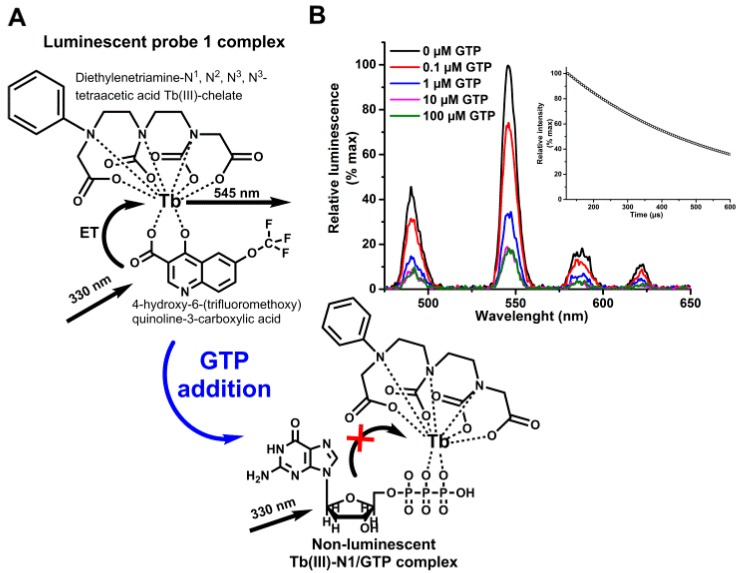
Apparent mode of action of the label-free nucleotide sensor. (**A**) Tb(III)-N1 forms a luminescent Probe 1 complex with antenna 1 in neutral or acidic aqueous solution. Addition of the phosphate-containing molecule, such as GTP, induce competitive antenna 1 replacement from the complex, forming non-luminescent Tb(III)-N1/GTP complex. (**B**) Luminescence emission and decay measurements showed typical Tb(III)-spectra and luminescent lifetime (inset) for Probe 1 (pH 7), responding negatively for increasing GTP concentration.

First, we selected functional terbium(III)-probe enabling phosphate content based separation of the studied molecules in neutral pH. The ion carrier (diethylenetriaminetetraacetic acid) is in a stable complex with Tb(III) forming Tb(III)-N1-chelate, which is itself incapable to sensitize the Tb(III)-luminescence without the antenna ligand, when 330 nm excitation light is used (Figure S1). With antenna, formed chelate-ligand complex gave the characteristic Tb(III) luminescence and luminescence lifetime of 590 ± 4 µs, enabling 330 nm excitation and time-gated measurements at the Tb(III) main peak of 545 nm in pH range from 6 to 8 (Figure 1, Figure S2). The Tb(III)-ion is strongly coordinated to the carrier ligand by seven donor atoms providing two coordination sites for the light harvesting antenna [25]. Phosphate-containing molecules can readily compete the antenna and occupy the two remaining coordination sites (Figure 1). In the presence of phosphate-containing nucleotide, competition between nucleotide and the antenna ligand induce the luminescence quenching, when antenna is displaced from the Probe 1 complex by phosphates of the nucleotide [26]. These interactions occur efficiently in neutral or slightly acidic buffer conditions, ideal for the most of the biologically relevant reactions (Figure S2).

**Figure 2:**
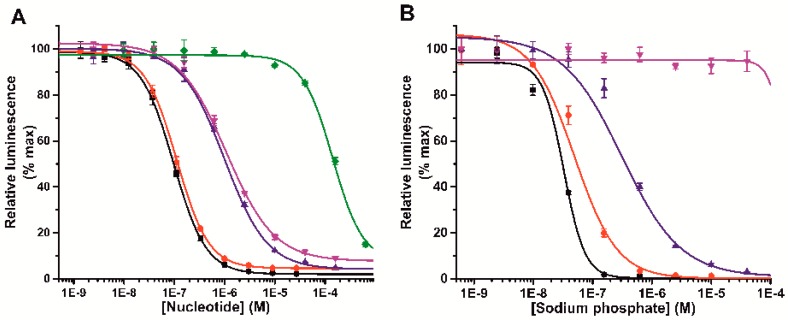
Time-gated Tb(III)-luminescence response curves of Probe 1 with different phosphate-containing molecules. (**A**) Probe 1 showed nanomolar nucleotide triphosphate detection sensitivity (GTP; black and ATP; red) and 10- to 1000-fold specificity over nucleotide di- and monophosphates (GDP; blue, ADP; magenta, and GMP; green), respectively. (**B**) Probe 1 detects sodium polyphosphates in similar phosphate number order as nucleotides, (NaPO_3_)_n_ (black) > (NaPO_3_)_3_ (red) > (NaPO_3_)_2_ (blue) >> Na_2_HPO_4_ (magenta). Data represent mean ± SD (n = 3).

To sensitize Tb(III)-ion luminescence, six different antenna molecules were first tested in the presence of different NTPs. All antennas (1o 6) sensitized Tb(III)-ion directly, but we found that complexes were sensitive to assay components such as enzymes and peptides (Table S1). Therefore, we selected 7-dentate Tb(III)-N1-chelate as a ion carrier to provide kinetic and thermodynamic stability to the probe, and to minimize the interferences from other ions and assay components, even the ultimate sensitivity was slightly compromised. In the selected buffer (pH 7), 4-hydroxy-6-(trifluoromethoxy)quinoline-3-carboxylic acid (antenna 1) showed the highest stability and robustness with relatively high signal-to-background ratio (S/B over 40). Also, nanomolar sensitivity for the sensor was observed when tested with or without GTP (Table S1, Figure S3). The antenna 1 and Tb(III)-N1-chelate complex was named as a Probe 1, used in all further assays. The ability of the Probe 1 to sense the number of phosphates was monitored with different phosphate containing anions. In a typical nucleotide titration, NTPs are detected in low nanomolar concentration, while nucleotide di- (NDP) and monophosphates (NMP) require 10 to 1000-fold higher concentrations, respectively (Figure 2a). For example, the limit-of-detection (LOD) for GTP, GDP, and GMP were 18.2 ± 2.5 nM, 197 ± 42 nM, and >15,000 nM, respectively. As expected, Probe 1 showed similar binding behavior independently of the nucleotide, solely separating the tested nucleotides by the number of phosphates. Cyclic nucleotides (cAMP and cGMP) were undetectable for the sensor. Phosphate dependence was further demonstrated by testing different polyphosphates, which quenched the luminescence in the following order (NaPO_3_)_n_ > (NaPO_3_)_3_ > (NaPO_3_)_2_ >> Na_2_HPO_4_ > NaH_2_PO_4_ (Figure 2b). This further confirms the Probe 1 ability to monitor directly the number of phosphates. Table 2 summarizes the detection properties of the Probe 1 with different phosphate-containing molecules.

### 3.2. K-Ras Functionality Can Be Monitored at Nanomolar Sensitivity Similar to the Control Assay

**Figure 3:**
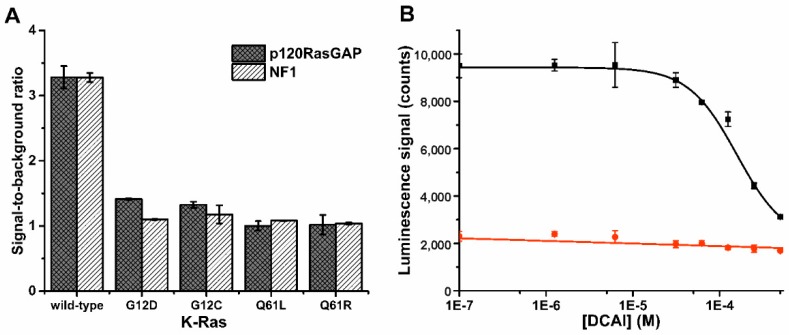
Time-gated Tb(III)-luminescence monitoring of the K-Ras GTPase activities. (**A**) GTPase cycle of GDP-GTP exchange (SOS^cat^) and GTP hydrolysis (p120RasGAP or NF1) with wild-type K-Ras reduces GTP concentration increasing Probe 1 time-gated Tb(III)-luminescence. This GTP hydrolysis in impaired with mutant K-Ras proteins, which gave low Tb(III)-luminescence. (**B**) DCAI inhibits SOS^cat^ dependent GDP-GTP exchange and thus impairs GTP hydrolysis resulting Tb(III)-luminescence quenching with wild-type K-Ras (black), while no change in Q61R K-Ras mutant (red) signal were detected. Data represent mean ± SD (n = 3).

Thereafter, we applied the Probe 1 in biological applications to demonstrate the concept functionality. First, we studied Ras activity with probe 1, and these assays were performed side-by-side with the previously introduced QRET assays used as a reference [22,23]. These assays were selected to enable direct comparison in a same buffer and assay conditions. We started by monitoring GTPase cycling, using K-Ras as an active GTP hydrolyzing enzyme (Figure S4a). K-Ras is frequently mutated in various cancers, and these mutations impair GTP hydrolysis leading to constitutive activation of the K-Ras downstream signaling [27,28]. Thus, K-Ras is a highly attractive protein as a drug target. In the assay, a guanine nucleotide exchange factor (SOS^cat^) and a GTPase activating proteins (NF1 or p120RasGAP) are required to catalyze GTP hydrolysis (Figure S4a). Only wild-type K-Ras, in comparison to the mutants (G12C, G12D, Q61L, and Q61R), showed increased luminescence due to the weaker luminescence quenching ability of GDP compared to GTP (Figure 3a, Figure S5). The functionality of the GTP hydrolysis assay was further tested by running a dose-response data on nucleotide exchange inhibitor (DCAI) [29]. This assay was also performed using previously described GTP-specific Fab-fragment based QRET assay (Figure 3b, Figure S6) [23,24]. In the assay with wild-type K-Ras, SOS^cat^, and p120RasGAP, the IC_50_ value monitored with DCAI and Probe 1 was 158 ± 13 µM, and the observed S/B ratio was 4.5 (Figure 3b). The QRET assay gave an IC_50_ value of 199 ± 8 µM with the S/B of 5.1 (Figure S7). In either assay, no change in signal was observed with a hydrolysis dead Q61R K-Ras mutant. As DCAI was originally reported as a nucleotide exchange inhibitor, we monitored the IC_50_ values also with K-Ras and SOS^cat^ in a nucleotide exchange assay (Figure S4b), taking into account that the GTP bound to the K-Ras cannot anymore induce the Tb(III)-luminescence quenching. The IC_50_ value of 232 ± 28 µM (S/B = 3.0) was monitored with Probe 1 (Figure S8) and 233 ± 22 µM (S/B = 10.3) with Eu(III)-GTP based QRET assay used as a reference method (Figure S9) [22,23]. The IC_50_ values measured for DCAI are similar to those reported previously [29].

### 3.3. Probe 1 Enables Nucleotide Triphosphate Hydrolysis Monitoring to Di- and Further to Nucleotide Monophosphates

**Figure 4:**
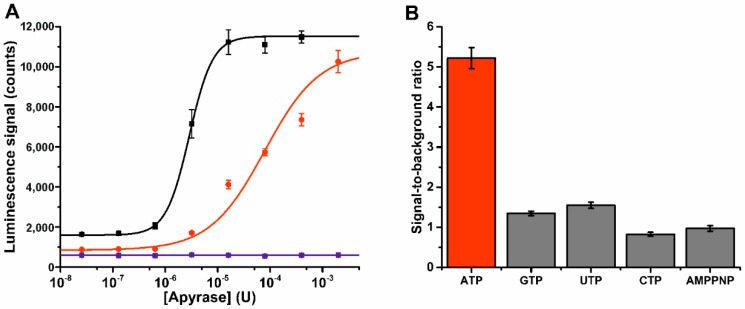
Time-gated Tb(III)-luminescence monitoring of apyrase ATPase activities. (**A**) Apyrase was titrated in the presence or 1 µM ATP (black), and 10 µM ATP (red) or with 10 µM AMP-PNP (blue), and ATPase activity was monitored from the increasing Tb(III)-luminescence signal. (**B**) Apyrase (50 µU) showed ATP specific hydrolysis activity, when assayed with 10 µM nucleotide triphosphate (ATP, GTP, UTP, CTP, and AMP-PNP). Data represent mean ± SD (n = 6).

Next, we assayed a panel of NTPs and NDPs with apyrase ATPase enzyme (Figure S4c). Apyrases are widely used tools in biotechnological applications, but they also possess important role in the maintenance of blood fluidity [30]. As a model, we selected potato apyrase and it was titrated at two fixed ATP concentration (1 µM or 10 µM) in the developed assay using Probe 1 (Figure 4a). Increased Tb(III)-signal indicates efficient ATP hydrolysis under the used assay conditions (buffer 2, pH 6.5). As expected, no signal change was monitored with non-hydrolysable ATP analog, AMP-PNP. This highlighted the relation between signal change and enzymatic ATP hydrolysis. Within 50 min incubation, full ATP hydrolysis using 1 µM and 10 µM of ATP was achieved with 16 µU (S/B 7.3) or 2 mU (S/B 11.5) of apyrase, respectively. It is known that different apyrases have different specificity patterns for nucleotides [30]. Thus we tested the ATP specificity of the used apyrase by performing the hydrolysis reaction at single NTP (10 µM) or NDP (50 µM) concentration and with a nucleotide panel (ATP, GTP, UTP, CTP, AMP-PNP, ADP, GDP, and UDP). Apyrase showed a clear ATP specificity in the NTP titration (Figure 4b), and only minor hydrolysis was detected with UTP and GTP, without detectable hydrolysis of CTP or AMP-PNP. However, in the NDP hydrolysis assay the adenosine nucleotide preference was less clear, and also GDP and UDP were hydrolyzed to corresponding NMPs (Figure S10). The observations in specificity might be due to the used apyrase, which is a mixture of two different apyrase species with varying specificity patterns and buffer preferences [31,32].

Here we presented homogeneous label-free method for sensitive NTP detection using two NTP processing model enzyme systems, K-Ras and apyrase. There is a constant need for improved NTP sensing and especially ATP monitoring is of high importance. However, most of the current methods are not simple and convenient to use. This is mostly because of laborious assay protocols with multiple different detection components and incubation steps [9]. Now introduced label-free assay enables enzyme activity monitoring and detection of small molecules modulating the enzyme activity. Assays can be performed simply in two steps, by first performing the enzyme reaction and thereafter adding the detection components. Low material consumption and costs are achieved with Tb(III)-probe enabling time-gated monitoring and thus low background interferences. Now introduced label-free methods showed equal performance as the previously developed single-label QRET assays for K-Ras monitoring. Tb(III)-probe, however, enables more versatile assays compared to QRET, thus the assay principle is directly applicable to monitor different NTP related reactions with purified enzymes other than Ras. This was demonstrated with apyrase NTP and NDP hydrolysis assays. It is expected that the Tb(III)-probe can be used also to monitor reactions producing NTPs. Also, assay functionality with more complex matrixes is yet to be resolved.

## 4. Conclusions

In conclusion, we have developed a label-free luminescent Tb(III)-probe for nucleotide polyphosphate detection with nanomolar sensitivity. The probe can distinguish NTPs from NDPs and NMPs in neutral aqueous solution, and the detection strategy was proved to be functional in monitoring of enzymatic activity of K-Ras and potato apyrase, and also in HTS compatible inhibitor studies. Probe 1 was shown to enable monitoring of highly relevant enzymatic reactions related to ATP and GTP biology, and thus it is expected to be suitable for a large variety of different reactions related to phosphate-containing molecules.

## Figures and Tables

**Table 1:** Summarized K-Ras GTPase cycling and GTP association and apyrase ATPase assay components.

Method	Detection	Reaction	Assay Buffer	Measurement
GTPase cycling assay	Probe 1: 7.5 µM antenna 1, 7.5 nM Tb(III)-N1 QRET: 7.5 nM Eu(III)-GTP, 12 nM 2A4^GTP^ Fab, 1.8 µM MT2	200 nM K-Ras, 200 nM SOS^cat^, 100 nM GAP (p120RasGAP or NF1), 1.5 µM GTP	Buffer 1: 20 mM HEPES, pH 7.5, 1 mM MgCl_2_, 0.01% Triton-X 100, 0.005% γ-globulins	Probe 1: 330/545 nm Tb(III)-luminescence (10 µL) QRET: 340/615 nm Eu(III)-luminescence (10 µL)
GTP association assay	Probe 1: 7.5 µM antenna 1, 7.5 nM Tb(III)-N1 QRET: 10 nM Eu(III)-GTP, 1.5 µM MT2	500 nM K-Ras, 250 nM SOS^cat^, 500 nM GTP 200 nM K-Ras, 200 nM SOS^cat^,	Buffer 1: 20 mM HEPES, pH 7.5, 1 mM MgCl_2_, 0.01% Triton-X 100, 0.005% γ-globulins	Probe 1: 330/545 nm Tb(III)-luminescence (10 µL) QRET: 340/615 nm Eu(III)-luminescence (10 µL)
Apyrase assay	Probe 1: 7.5 µM antenna 1, 7.5 nM Tb(III)-N1	50 µU apyrase, 1–10 µM ATP	Buffer 2: 20 mM HEPES, pH 6.5, 1 mM MgCl_2_, 1 mM CaCl_2_, 0.01% Triton-X 100, 0.005% γ-globulins	Probe 1: 330/545 nm Tb(III)-luminescence (10 µL)

**Table 2:** Summary of the binding properties of the Probe 1 to different phosphate-containing anions ^a^.

Anion Species	EC_50_ (nM)	LOD (nM)	Linear Range (µM)
ATP	107 ± 0.5	15.7 ± 3.0	0.02–5
GTP	97 ± 0.4	18.2 ± 2.5	0.02–10
CTP	17 ± 0.2	3.1 ± 1.5	0.01–1
ITP	155 ± 0.4	43.6 ± 5.7	0.02–10
UTP	164 ± 0.7	28.2 ± 4.4	n.c.
AMP-PNP	73 ± 0.6	8.6 ± 3.9	n.c.
ADP	1170 ± 70	161 ± 48	0.2–150
GDP	1110 ± 60	197 ± 42	0.2–100
UDP	1540 ± 70	165 ± 36	0.2–150
GMP	16,800 ± 3900	n.c.	n.c.
(NaPO_3_)_n_	33 ± 0.2	13.6 ± 7.2	n.c.
(NaPO_3_)_3_	60 ± 0.4	15.5 ± 2.6	n.c.
(NaPO_3_)_2_	432 ± 3	69.3 ± 20.2	n.c.
Na_2_HPO_4_	>250	n.c.	n.c.
cGMP	n.d.	n.d.	n.d.
cAMP	n.d.	n.d.	n.d.

^a ^Experimental conditions: excitation 330 nm, emission 545 nm, delay 100 µs, decay 200 µs, [Tb(III)-N1] = 7.5 nM, [4-hydroxy-6-(trifluoromethoxy)quinoline-3-carboxylic acid] = 7.5 µM, Assay buffer 1 [20 mM HEPES, pH 7, 1 mM MgCl_2_, 0.01% Triton-X 100, 0.005% γ-globulins], error represents SD, n = 3 or n = 5. n.d. not detected. n.c. not calculated.

## Supplementary Materials

The following are available online at http://www.mdpi.com/1424-8220/18/11/3989/s1, Supplementary Figures S1–S10 and Table S1.
