# Supplementary material for: Label-Free Time-Gated Luminescent Detection Method for the Nucleotides with Varying Phosphate Content
Source: Sensors (Basel). 2018 Nov 16;18(11):3989. doi: 10.3390/s18113989 (PMC6264117; doi:10.3390/s18113989)
Supplement: Supplementary file 1 [file sensors-18-03989-s001.pdf]

Label-Free Time-Gated Luminescent Detection Method for the Nucleotides with Varying Phosphate Content

Kari Kopra,[1,*] Tanja Seppälä,[1] Dana Rabara,[2] Maria Abreu-Blanco,[2] Sakari Kulmala,[3] Matthew Holderfield,[2] and Harri Härmä[1]

[1] Materials Chemistry and Chemical Analysis, University of Turku, Finland

[2] NCI-RAS Initiative, Cancer Research Technology Program, Frederick National Laboratory for Cancer Research, Leidos Biomedical Research, USA

[3] Laboratory of Analytical Chemistry, Aalto University, Finland

Corresponding Author: kari.kopra@utu.fi

SUPPORTING INFORMATION

Table of contents

Supporting results

**Fig. S1-S3, Table S1**	**Probe 1 selection, functionality, and reproducibility**
**Fig. S4**	**Principles of the enzymatic reactions**
**Fig. S5-S9**	**Probe 1 and QRET functionality in enzymatic K-Ras assays**
**Fig. S10**	**Probe 1 functionality in apyrase inhibition assay**

Supporting references

Supporting results

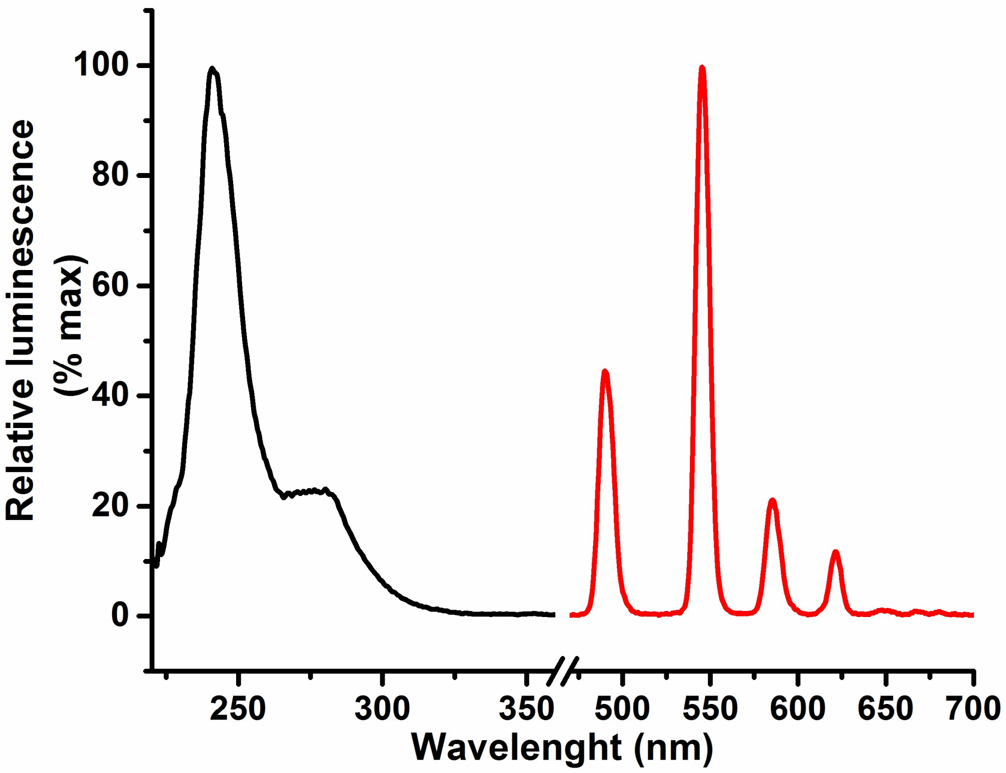

Fig. S1 Tb(III)-N1-chelate excitation and emission spectra without light harvesting antenna. Tb(III)-N1-chelate excitation (220-400 nm) and emission (400-700 nm) spectra's were measured in the assay buffer 1 (pH 7). Tb(III)-N1-chelate can be excited directly using high energy UV-light (excitation maximum at 240 nm), providing characteristic Tb(III) emission spectra. However, 330 nm excitation wavelength used in the developed assay is not suitable for Tb(III)-N1-chelate excitation without light harvesting antenna. Emission and excitation are separately normalized to Tb(III)-N1-chelate intensity maximum.

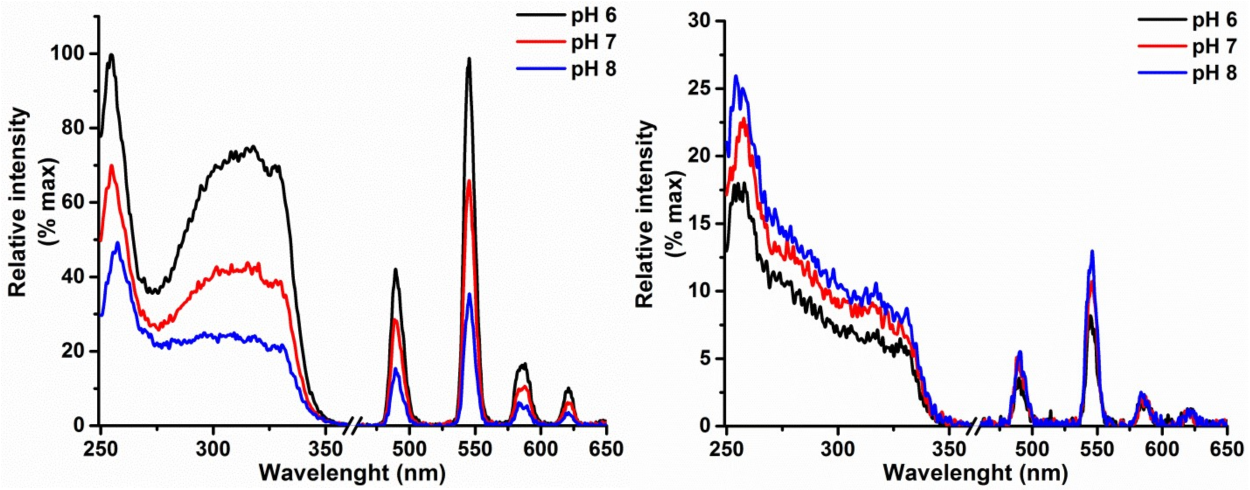

Fig. S2 Probe 1 excitation and emission spectra at three different pH. Probe 1 excitation (220-400 nm) and emission (400-700 nm) spectra's were measured in the assay buffer 1 set in pH ranging from 6 to 8. Probe 1 responded to pH preferring slightly acidic conditions. Maximum signal is monitored in assay buffer pH 6 (left). Also the response to ATP (10 μM) addition is most prominent in the buffer with pH 6 (right). Data indicates that Probe 1 is not efficiently formed in basic solution, but instead prefers neutral or acidic conditions. Emission and excitation are separately normalized to Probe 1 intensity maximum at pH 6.

Table S1 Functionality of the antenna ligands 1-6 with $TbCl_3$ and Tb(III)-N1.[a]

		**7.5 nM $TbCl_3$**			**7.5 nM Tb(III)-N1**		
**Antenna ligands**	**Structure**	**S/B, 5 min**	**S/B, 30 min**	**S/B, 90 min**	**S/B, 5 min**	**S/B, 30 min**	**S/B, 90 min**
**1,** 4-hydroxy-6-(trifluoromethoxy)-quinoline-3-carboxylic acid	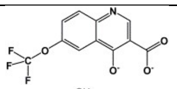	**55**	**56**	**50**	**26**	**48**	**47**
**2,** 4-hydroxy-7-methyl-1,8-naphtyridine-3-carboxylic acid	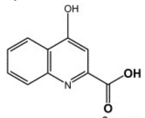	**69**	**67**	**66**	**42**	**62**	**76**
**3,** 1-cyclopropyl-6-fluoro-4-oxo-7-piperazin-1-yl quinoline-3-carboxylic acid	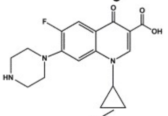	**5.6**	**4.5**	**5.1**	**3.7**	**3.7**	**3.6**
**4,** 1-ethyl-1,4-dihydro-7-methyl-4-oxo-1,8-naphthyridine-3-carboxylic acid	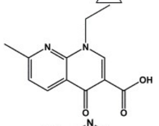	**109**	**112**	**93**	**45**	**105**	**113**
**5,** 2-methyl-7-oxo-4,7-dihydropyrazolo[1,5-a]pyrimidine-6-carboxylic acid	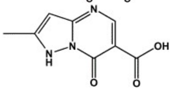	**25**	**20**	**22**	**15**	**26**	**33**
**6,** 4-oxo-1 4-dihydroquinoline-3-carboxylic acid	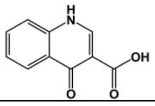	**52**	**57**	**53**	**27**	**48**	**44**

[a] Experimental conditions: excitation 330 nm, emission 545 nm, delay 100 µs, decay 200 µs, [Tb(III)-N1 or $TbCl_3$] = 7.5 nM, antenna 1-6 = 7.5 µM, GTP = 10 µM, assay buffer 1 [20 mM HEPES, pH 7.5, 1 mM $MgCl_2$, 0.01 % Triton-X 100, 0.005 % γ-globulins], n = 3.

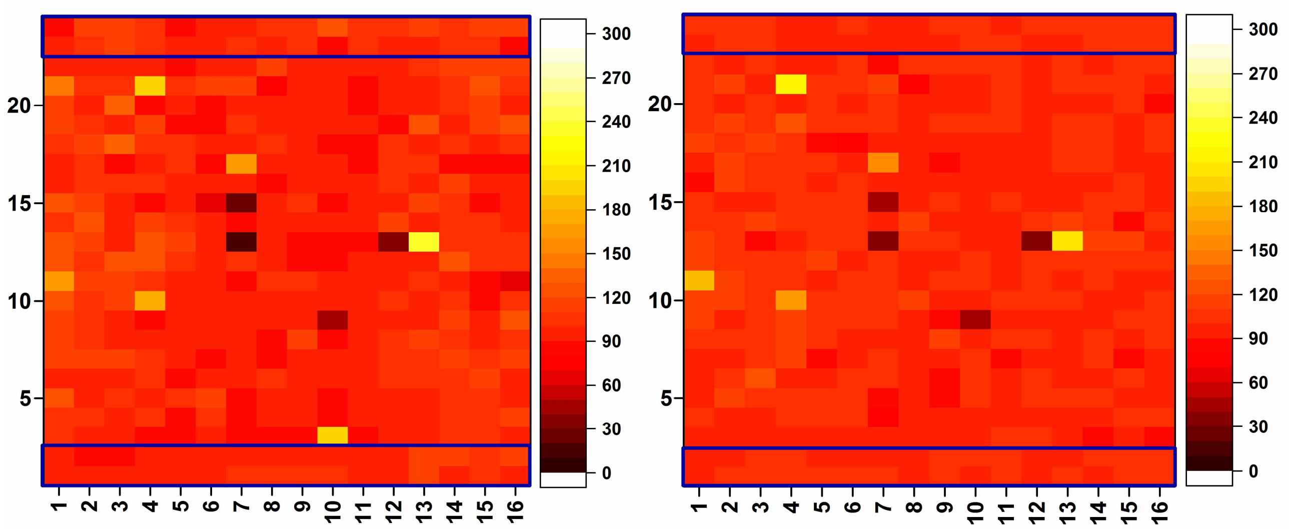

Fig. S3 Library compound test with Probe 1. Reproducibility and robustness of the assay with Probe 1 was determined with 320 randomly selected small molecule library compounds (20 µM) with (left) or without (right) 1.5 µM GTP. Used 64 DMSO control wells are lined around in blue. From the 64 controls, we calculated the positive and negative cutoffs (3SD). Using these cutoffs, 2.2-2.5% of compounds increased and 1.9-2.2% decreased the Tb(III)-signal over ±3SD cutoff. Thus these compounds can be referred as interference in GTP monitoring assay performed with Probe 1. The Z'-factor calculated from the controls in the plate 1 and 2 was 0.82. Luminescence signals are normalized to Probe 1 average intensity in DMSO control wells.

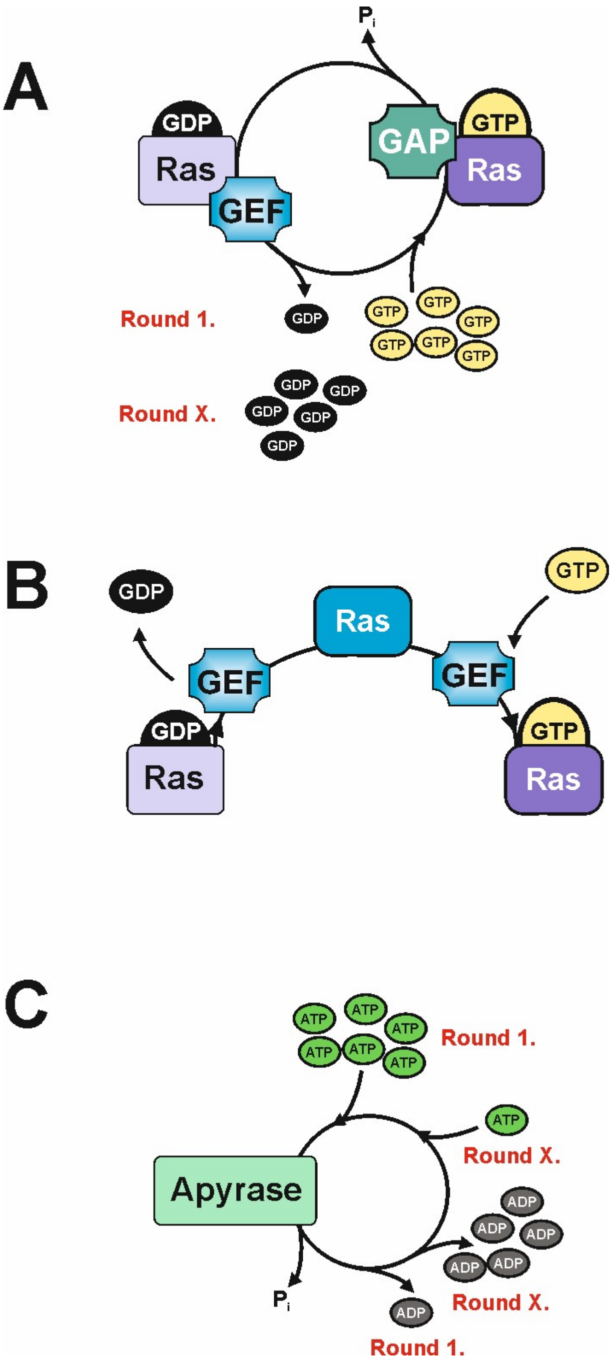

Fig. S4 Model enzymatic reactions monitored using Probe 1. A) In the GTPase cycling assay (K-Ras), GTP hydrolysis in the presence of GEF and GAP reduces free GTP concentration by hydrolyzing it to phosphate and GDP. This enzymatic GTP hydrolysis was observed from increasing Probe 1 signal. Similarly, GTP hydrolysis was observed from increasing QRET signal in the reference assay, using GTP specific Fab fragment and Eu(III)-GTP [1]. B) In the GTP association assay with Ras, the GTPase bound GDP is dissociated in the presence of GEF, enabling GTP association. The reduced free GTP concentration is observed from increasing Probe 1 signal. Direct Eu(III)-GTP association was used as a reference method [1,2]. C) In the apyrase assay, ATP is hydrolyzed to phosphate and ADP. Using Probe 1, reduced ATP concentration can be observed from increasing signal. Apyrase can also hydrolyze other nucleotide di- and triphosphates than ATP, and this can be similarly monitored using Probe 1.

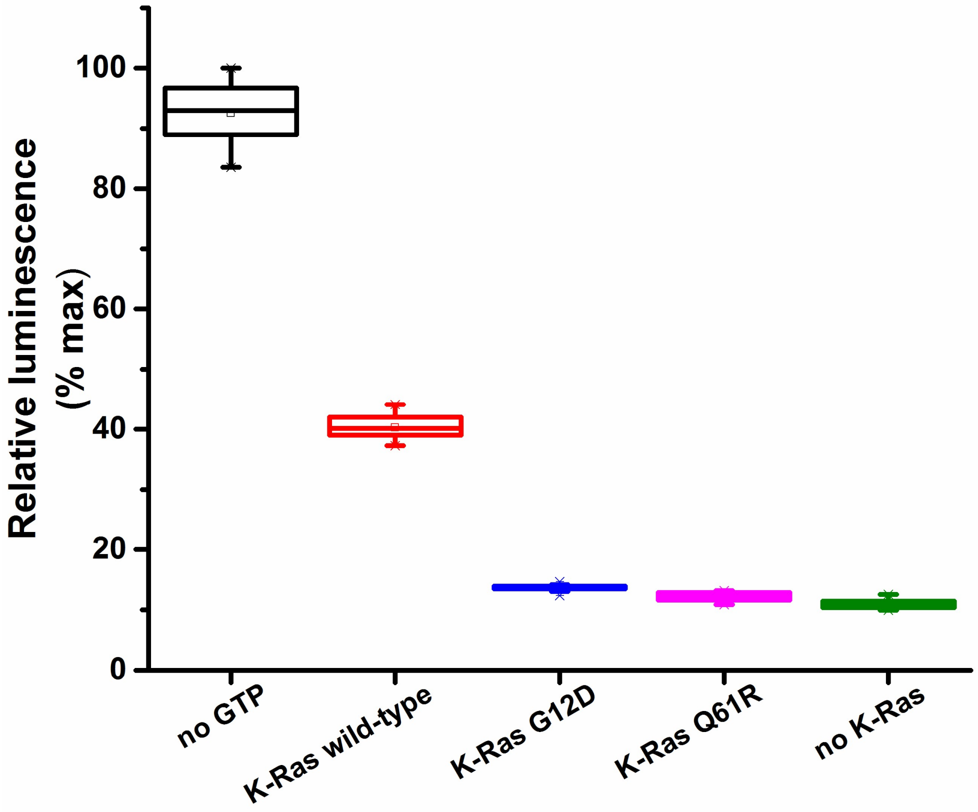

Fig. S5 Reproducibility of the GTPase cycling assay performed with Probe 1. GTPase cycling assay was performed with wild-type K-Ras and with two K-Ras mutants (G12D and Q61R). As a positive and negative control we used reactions without GTP or K-Ras. In all assays, K-Ras and SOS^{cat} were used in 200 nM, p120RasGAP in 100 nM, and GTP in 1.5 µM concentration. Time-gated Tb(III)-signals were monitored 60 min after Probe 1 addition. High reproducibility and HTS suitability was monitored for the GTPase cycling assay, when K-Ras wild-type was compared with the two K-Ras mutants (Z'-factor 0.72-0.73). Data represent mean ± SD (n=24).

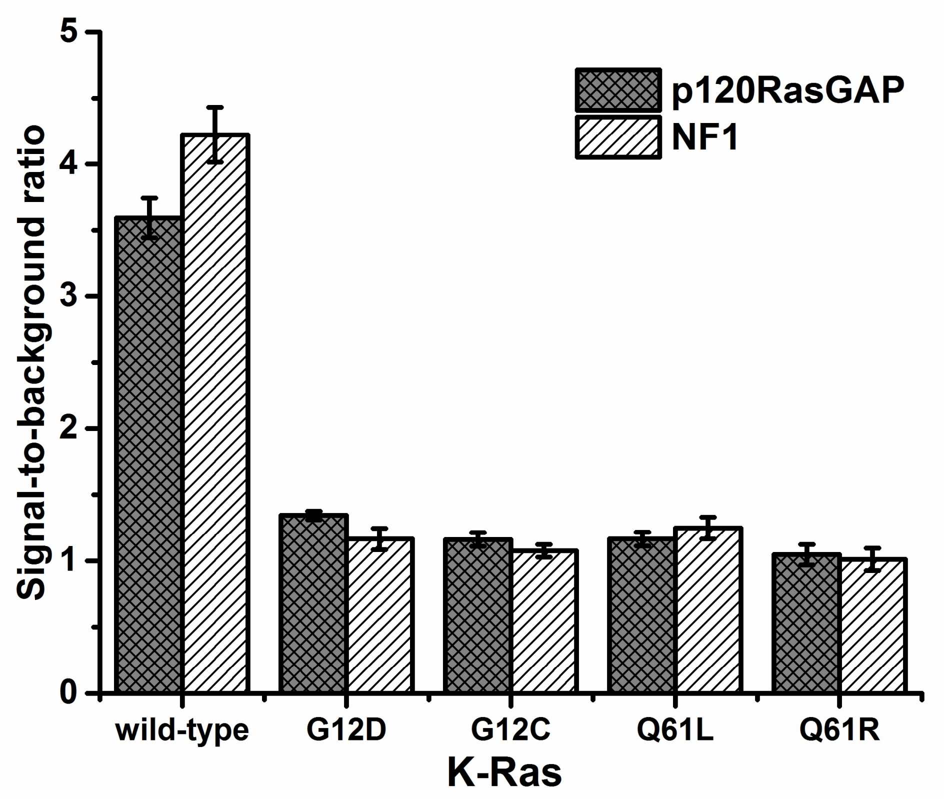

Fig. S6 QRET based GTPase cycling monitoring. In the assay, GTPase cycle of GDP-GTP exchange and GTP hydrolysis with wild-type K-Ras reduces free GTP concentration, which increases Eu(III)-GTP binding to GTP-specific $2A4^{GTP}$ Fab fragment. GTP hydrolysis is impaired for mutant K-Ras proteins (G12D, G12C, Q61L, and Q61R), resulting in low Eu(III)-luminescence signal due to compatition. Assay was performed using 200 nM K-Ras, 1.5 μM GTP, 200 nM SOS^{cat}, and either with 100 nM p120RasGAP (dark gray) or 100 nM NF1 (light gray). The detection solution added after 60 min reaction contains 7.5 nM Eu(III)-GTP, 12 nM $2A4^{GTP}$, and 1.8 μM MT2. Signal-to-background ratio (S/B) with wild-type K-Ras and K-Ras mutants (G12D, G12C, Q61L, and Q61R) using p120RasGAP were 3.6, 1.3, 1.2, 1.2, and 1.0, respectively. Similarly, with NF1 the S/B ratios were 4.2, 1.2, 1.1, 1.2, and 1.0 for wild-type K-Ras and K-Ras mutants (G12D, G12C, Q61L, and Q61R), respectively. Data represent mean ± SD (n=3) at a time point of 15 min.

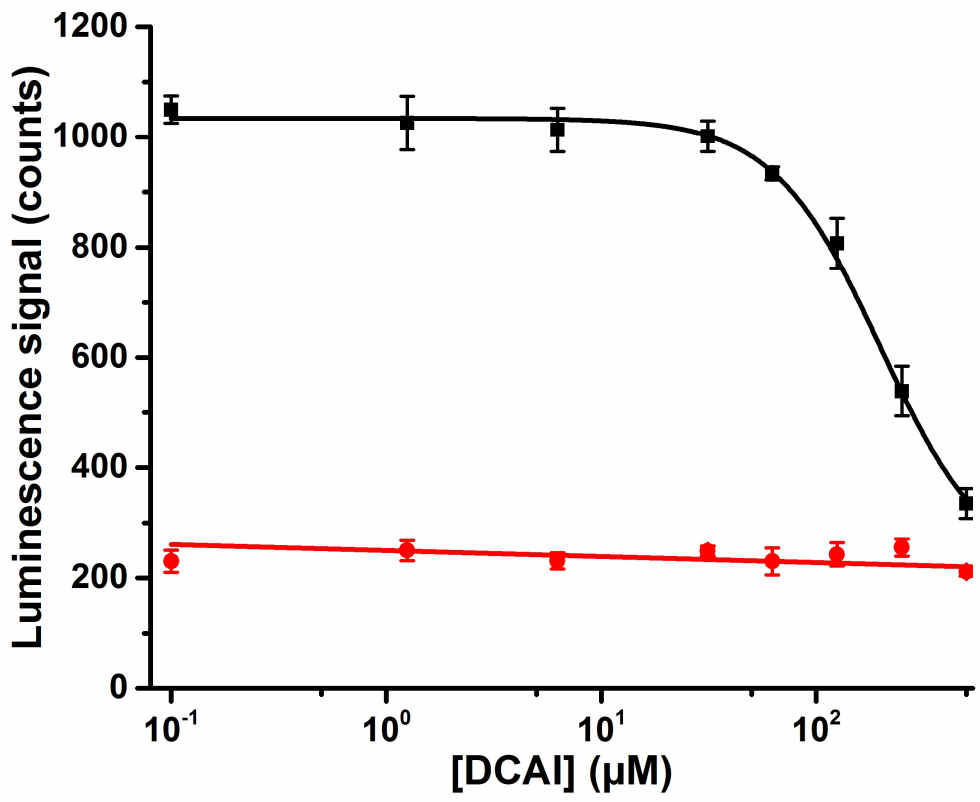

Fig. S7 DCAI titration using QRET based GTPase cycling assay. In the GTPase cycling assay, wild-type K-Ras reduces free GTP concentration, which increases Eu(III)-GTP binding to $2A4^{GTP}$ Fab fragment. DCAI inhibits SOS^{cat} dependent GDP-GTP exchange and thus impairs GTP hydrolysis, resulting in low Eu(III)-luminescence signal due to blocked GTP hydrolysis. Assay was performed using either 200 nM K-Ras wild-type (black) or 200 nM K-Ras Q61R mutant (red), and with 200 nM SOS^{cat}, 100 nM p120RasGAP, and 1.5 µM GTP. Detection solution contain 7.5 nM Eu(III)-GTP, 12 nM $2A4^{GTP}$, and 1.8 µM MT2. S/B ratios for wild-type and Q61R mutant K-Ras were 5.1 and 1.1, respectively. The Q61R mutant K-Ras is hydrolysis dead and thus does not respond to DCAI concentration. In DCAI titration (1.25-500 µM) the calculated IC_{50} value with wild-type K-Ras was 199 ± 8 µM [3]. Data represent mean ± SD (n=3) at a time point of 15 min.

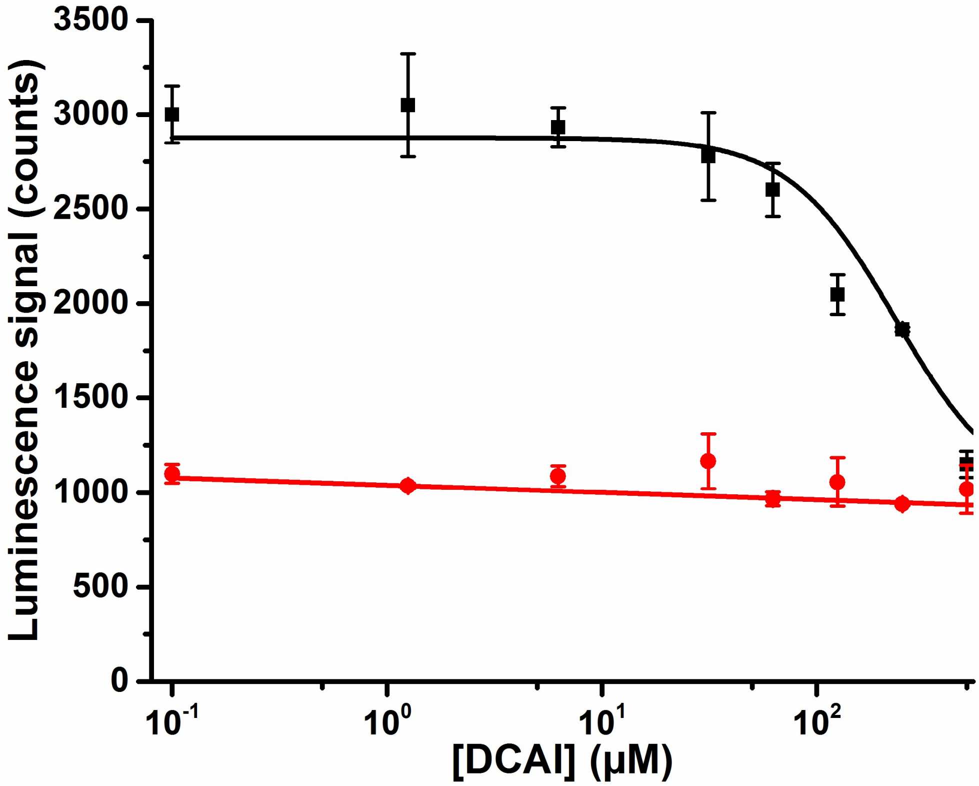

Fig. S8 DCAI titration using K-Ras nucleotide association assay performed with Probe 1. DCAI titration (1.25-500 μM) was performed to monitor 500 nM GTP association inhibition to 500 nM K-Ras (wild-type or Q61R mutant) in the presence of nucleotide exchange factor (SOS^{cat}). Probe 1 monitored GTP, which in the absence of DCAI is bound to K-Ras and its ability to quench the Probe 1 luminescence is impaired. DCAI blocks the GTP association and thus GTP can compete the binding of antenna-ligand in the Probe 1 complex. The calculated IC_{50} value with wild-type K-Ras (black) was 232 ± 28 μM (S/B = 3.0), while no change in Q61R K-Ras mutant (red) signal was detected due to impaired GTP association [1]. Data represent mean ± SD (n=3).

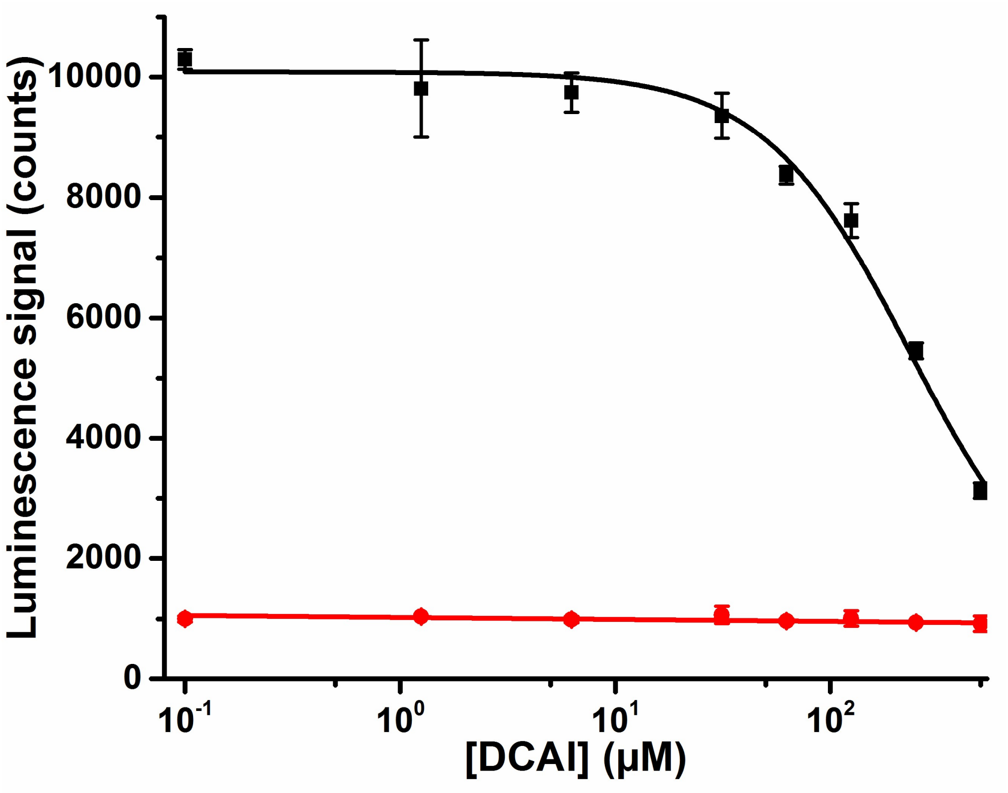

Fig. S9 DCAI titration using QRET based Eu(III)-GTP association assay. In the assay without inhibitor compound, Eu(III)-GTP association increases Eu(III)-luminescence signal due to K-Ras ability to protect the Eu(III)-signal from the MT2 induced quenching. In the presence of DCAI, Eu(III)-GTP association cannot occur and low Eu(III)-luminescence signal is monitored due to high quenching of the Eu(III)-GTP signal in solution by MT2. Assay was performed using either 200 nM K-Ras wild-type (black) or 200 nM K-Ras Q61R mutant (red), and with 200 nM SOS^{cat}, 7.5 nM Eu(III)-GTP, and 1.5 μM MT2. S/B ratios with wild-type K-Ras and Q61R mutant were 10.3 and 1.1, respectively. In DCAI titration (1.25-500 μM) the calculated IC_{50} value with wild-type K-Ras was 233 ± 22 μM [1]. Data represent mean ± SD (n=3) in a time point of 30 min.

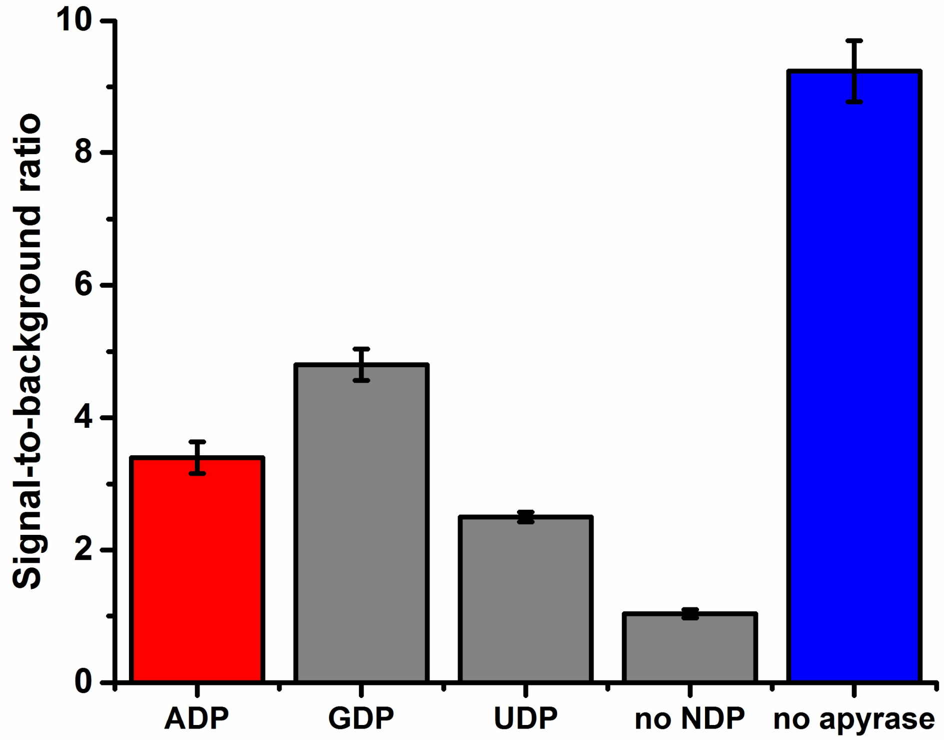

Fig. S10 Apyrase ability to hydrolyze different nucleotide diphosphates (NDPs). Apyrase ADPase activity and specificity were monitored with single enzyme (200 µU) and NDP (50 µM) concentrations by using Probe 1 for detection. Enzymatic reaction was incubated for 50 min before detection. Apyrase showed no ADP specificity, but were able to hydrolyze also two other tested NDPs (GDP and UDP). Thus similar nucleotide preference as with NTPs were not monitored with NDPs. Data represent mean ± SD (n=3) in a time point of 30 min.

Supporting references

1. Kopra, K.; van Adrichem, A.J.; Salo-Ahen, O.M.H.; Peltonen, J.; Wennerberg, K.; Härmä, H. High-throughput dual screening method for Ras activities and inhibitors. *Anal Chem.* **2017**, *89*, 4508–4516.
2. Kopra, K.; Ligabue, A.; Wang, Q.; Syrjänpää, M.; Blaževitš, O.; van Adrichem, A.J.; Hänninen, P.; Veltel, S.; Abankwa, D.; Härmä, H. A homogeneous quenching resonance energy transfer assay for the kinetic analysis of the GTPase nucleotide exchange reaction. *Anal. Bioanal. Chem.* **2014**, *406*, 4147–4156.
3. Maurer, T.; Garrenton, L.S.; Oh, A.; Pitts, K.; Anderson, D.J.; Skelton, N.J.; Fauber, B.P.; Pan, B.; Malek, S.; Stokoe, D.; Ludlam, M.J.; Bowman, K.K.; Wu, J.; Giannetti, A.M.; Starovasnik, M.A.; Mellman, I.; Jackson, P.K.; Rudolph, J.; Wang, W.; Fang, G. Small-molecule ligands bind to a distinct pocket in Ras and inhibit SOS-mediated nucleotide exchange activity. *Proc. Natl. Acad. Sci. U.S.A.* **2012**, *109*, 5299–5304.
